# CB-MTE: Social Bot Detection via Multi-Source Heterogeneous Feature Fusion

**DOI:** 10.3390/s25113549

**Published:** 2025-06-04

**Authors:** Meng Cheng, Yuzhi Xiao, Tao Huang, Chao Lei, Chuang Zhang

**Affiliations:** 1 School of Computer Science, Qinghai Normal University, Xining 810008, China; 202333331070@stu.qhnu.edu.cn (M.C.); 202133331057@stu.qhnu.edu.cn (T.H.); 202333331075@stu.qhnu.edu.cn (C.L.); 202333331046@stu.qhnu.edu.cn (C.Z.); 2 The State Key Laboratory of Tibetan Intelligence, Qinghai Normal University, Xining 810008, China

**Keywords:** social bot detection, heterogeneous feature fusion, graph embedding, DistilBERT, manifold learning, CB-MTE

## Abstract

Social bots increasingly mimic real users and collaborate in large-scale influence campaigns, distorting public perception and making their detection both critical and challenging. Traditional bot detection methods, constrained by single-source features, often fail to capture the complete behavioral and contextual characteristics of social bots, especially their dynamic behavioral evolution and group coordination tactics, resulting in feature incompleteness and reduced detection performance. To address this challenge, we propose CB-MTE, a social bot detection framework based on multi-source heterogeneous feature fusion. CB-MTE adopts a hierarchical architecture: user metadata is used to construct behavioral portraits, deep semantic representations are extracted from textual content via DistilBERT, and community-aware graph embeddings are learned through a combination of random walk and Skip-gram modeling. To mitigate feature redundancy and preserve structural consistency, manifold learning is applied for nonlinear dimensionality reduction, ensuring both local and global topology are maintained. Finally, a CatBoost-based collaborative reasoning mechanism enhances model robustness through ordered target encoding and symmetric tree structures. Experiments on the TwiBot-22 benchmark dataset demonstrate that CB-MTE significantly outperforms mainstream detection models in recognizing dynamic behavioral traits and detecting collaborative bot activities. These results confirm the framework’s capability to capture the complete behavioral and contextual characteristics of social bots through multi-source feature integration.

## 1. Introduction

The widespread adoption of digital technologies has reshaped the way human society is connected. The ITU released the Facts and Figures 2024 report, which predicts that by the end of 2024, the number of Internet users worldwide will reach 5.5 billion. With the popularity of the mobile Internet and the deepening of the functions of social media platforms, the Internet has become the main channel for information acquisition and social interaction. However, a large number of social bots are threatening the security of cyberspace through intelligent means. Social bots have evolved into agents capable of identity camouflage, behavior simulation, and group cooperation, which seriously damages the ecological balance of the network by means of spamming junk information and public opinion manipulation [1,2,3]. Such technological misuse poses a severe challenge to the existing detection system, and it is urgent to build a multi-source intelligent detection framework to cope with the rapidly evolving situation.

Social bot detection faces two core challenges: efficient identification of massive data and adaptation to dynamic evolutionary behavior. In traditional methods, explicit indicators such as user activity, user portraits, and text features are constructed, and algorithms such as Random Forest and Support Vector Machine are used to establish classification models [4,5]. Although it can effectively identify the programmed behavioral features of early bots, there are two fundamental defects: First, these methods are highly dependent on the features designed by humans, and it is difficult to cope with the feature camouflage attacks. Second, although the text analysis method based on NLP can extract semantic features [6], it faces text noise interference and insufficient semantic generalization caused by the content manipulation strategies.

In order to overcome these limitations, recent research has shifted to graph-based analysis, which provides a novel methodology for bot detection. Its core value lies in the in-depth mining of group behavior patterns. By constructing a user interaction graph, the topological features and propagation patterns of bot clusters can be effectively captured [7]. Furthermore, dynamic community division technology supported by structural information theory can identify abnormal group behaviors [8]. However, the prior art still faces two major constraints: First, the sparsity of social network structural data leads to incomplete feature extraction. Second, there is a significant contradiction between the real-time computing demands brought about by the evolution of dynamic networks and the static processing mechanism of existing models.

To sum up, it is difficult for traditional feature engineering to effectively resist feature camouflage attacks; text analysis is limited in identifying the content manipulation strategies; graph structure methods are often limited by structural data sparsity. Therefore, multi-source heterogeneous feature fusion and dynamic behavior perception have become the inevitable evolution direction of social bot detection.

We propose innovative solutions for the detection task of social bots. Core contributions are as follows:

(1) Multi-source heterogeneous feature collaborative modeling

To address the characterization limitations of single-source features in social bot detection, we propose a metadata-text-social topology multimodal collaborative analysis framework. Through innovative design of ten new metadata features, including device entropy, tweet mutation rate and other dimensions, the multi-granularity behavior profile system is constructed, and the semantic discrete features of the text are extracted by combining lightweight pre-training language models. The topological anomaly patterns in community communication are extracted by deep graph embedding techniques, and the influence difference of nodes is quantified by introducing multi-scale network centrality indices. A cross-modal feature space is then constructed through joint optimization.

(2) Hierarchical double fusion detection framework CB-MTE

We propose CB-MTE, a two-tier fusion framework that synergizes feature-level and model-level integration. At the feature level, the heterogeneous feature space mapping is realized by nonlinear dimensionality reduction algorithms based on manifold learning to solve the dimension mismatch problem of metadata, text and graph embeddings. At the model level, a dynamic weight allocation mechanism is constructed using the CatBoost gradient boosting tree, and the classification boundary is optimized by combining ordered target encoding, which significantly improves the detection accuracy compared to traditional methods.

(3) Fine-grained evaluation system

We established a cross-scenario evaluation benchmark using the TwiBot-22 dataset. To systematically validate framework robustness, five social sub-datasets covering political, entertainment, medical, and other domains were selected for cross-topic migration testing. Experimental verification showed that the macro F1 value of the framework reached 80.84% under the scenario of new topic camouflaging attack and dynamic evolution of social network structures. Compared with the mainstream model BotRGCN, it is 23.34 percentage points higher, and its performance indicators are significantly superior to those of mainstream baseline methods, which confirms the strong adaptability of the multi-source feature fusion mechanism to the complex evolution situation.

## 2. Related Work

### 2.1. Social Bot Detection

Social bots are automated accounts controlled by programs, and their behavior patterns are highly organized. Although some service bots exist, most of them are used in malicious activities such as forging interactive data, spreading junk information and manipulating public opinion [9]. In response to such threats, the current detection technology mainly adopts three types of technical routes: research paradigms based on feature engineering, text semantic analysis, and graph structure mining.

Feature-based approach: Relevant features are extracted from user metadata and published tweets, and then combined with a traditional binary classification model for bot detection [10]. Typical studies include Echeverria et al. [11], who developed an adaptive classifier for 20 new bot types through cross-class generalization tests; Alarfaj et al. [12], who integrated message features, part-of-speech tagging, and emotion polarity to construct feature sets to achieve fine-grained detection; Abreu et al. [13], who obtained real dataset from tweets, extracted and selected five core features, and used four types of machine learning models for training and evaluation.

Text-based approach: This path focuses on the content analysis of tweets, mining the semantic features, and generation patterns of bot texts. Wang et al. [14] used LSA model to extract four similarities of tweets for social bot detection of tweets. Duki et al. [6] used BERT-base model and a number of labels for bot detection according to the content of tweets. Kumar et al. [15] classify tweets as bot tweets or non-bot tweets based on the text content of the tweets.

Graph structure-based approach: This type of approach reveals group behavior characteristics by modeling user interaction networks. Feng et al. [7] capture topological patterns of bot interactions by constructing heterogeneous graph neural networks. Yu et al. [16] proposed a multi-modal detection framework based on social relationship subgraphs, which constructed user interaction subgraphs and linear splicing of text content features and behavioral gene sequences to generate social behavior features for bot recognition. Lin et al. [17] jointly encode user semantics, attributes, and neighborhood information, and adopt an improved graph attention network model to carry out parallel computation of large-scale graphs through subgraph sampling to realize bot detection.

Although the above methods have achieved remarkable results in their respective fields, the single detection paradigm has significant shortcomings in complex adversarial scenarios; for example, feature engineering is vulnerable to reverse engineering; text analysis is difficult to distinguish semantic confusion, and the graph structure relies on the complete social topology. Therefore, multi-source heterogeneous feature fusion has become a key direction to break through the limitations. In terms of multi-source feature fusion, Lei et al. [18] combined text and graph structure to recognize bots; Kudugunta et al. [19] use tweet content and metadata to detect Twitter bots, but there is still insufficient coverage of feature sources. Therefore, we propose a CB-MTE framework and use the triple collaborative mechanism of metadata behavior modeling, deep semantic understanding, and graph relationship inference to realize accurate identification of social bots.

### 2.2. Social Bot Detection Technology

#### 2.2.1. Text Modeling

In social media text analysis, BERT is widely used for feature extraction of user-generated content, such as tweets, profiles, and interactive comments [20]. Although existing studies have explored the potential of BERT in social media analysis, such as generating tweet embedment [21], capturing bot features in sentiment classification [22], and heterogeneous graph neighbor aggregation [23], problems such as relying on a single text mode and neglecting metadata fusion are generally found. Although the pre-trained language model based on Transformer improves the semantic understanding accuracy through bi-directional context coding [24], its huge number of parameters and high computing cost seriously restrict the deployment feasibility in real-time streaming data scenarios. In view of this core contradiction, we use DistilBERT, a lightweight semantic modeling framework compressed by BERT model knowledge distillation technology, which is smaller, faster, and lighter. The framework maintains 97% of the original semantic understanding performance while reducing the parameter number by 40%, and achieves a 60% improvement in reasoning speed [25], which is critical for processing massive streams of tweets. We use DistilBERT to dynamically encode text, integrate user metadata and social topology, and realize multi-dimensional collaborative enhancement.

#### 2.2.2. Graph Structure Embedding

Social bots often evade detection through star topology or community infiltration, and we use DeepWalk’s random walk strategy to model local structural features [26], and its truncated walk can effectively model these two attack modes. The walk path of the center node in the star topology presents high repeatability, while the walk sequence of the penetration bot shows cross-community migration characteristics. Compared with the homogeneity bias of Node2Vec [27] and the computational complexity of the Struc2Vec’s global structure [28], DeepWalk is simpler to implement and faster to process in large-scale graphs. In addition, Berriche et al. [29] proposed a hybrid approach that integrates DeepWalk to learn low-dimensional structural embeddings, thereby reducing computational complexity and enhancing robustness in complex networks. By further incorporating Beam Search, their method achieves significant improvements in both efficiency and robustness for community detection. Leskovec et al. [30] obtained graph embedding by using node feature information and structure information, which has outstanding performance in node classification and link prediction. In our work, we further enhance DeepWalk embeddings by integrating classical centrality measures and reducing dimensions via UMAP, yielding a compact and structurally informative feature representation.

#### 2.2.3. Decision Classification

We adopt CatBoost, a categorical gradient boosting algorithm [31], for multi-source feature classification. Compared with the traditional gradient boosting algorithms, CatBoost can process the original category data directly without the need for one-hot encoding when processing categorical features, thus avoiding the problems of data bloat and information loss. For example, Zhang et al. [32] used the bag-of-words approach to process TF-IDF for feature extraction and integrated the CatBoost algorithm for training, achieving a spam detection accuracy rate of over 98%. Ibrahim et al. [33] compared the performance of CatBoost with other classifiers on business process datasets, and found that CatBoost outperformed the alternatives. In our framework, CatBoost serves as the final decision layer, effectively integrating metadata, textual, and graph-based features.

## 3. Framework and Methods

### 3.1. Framework Architecture

**Figure 1:**
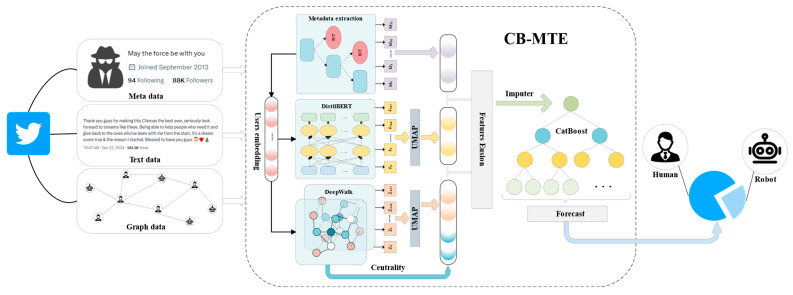
CB-MTE: Multi-source heterogeneous feature fusion framework.

Figure 1 shows an overview of the proposed CB-MTE framework, which consists of four modules that work together to improve the robustness and accuracy of social bot detection:

(1) Metadata feature extraction module: We extract user metadata from the TwiBot-22 dataset, including account attributes, behavioral attributes, and social attributes. Metadata is cleaned and standardized to eliminate noise and handle missing values. We then compute statistical and derived features based on prior work on behavioral profiling, and generate user embeddings to support downstream modules.

(2) Text Feature Extraction Module: We collect user-generated text and adopt DistilBERT [25] to produce high-dimensional, context-aware semantic embeddings. To reduce dimensionality while preserving structure, we apply UMAP (Uniform Manifold Approximation and Projection) [34], which has shown effectiveness in retaining global and local relationships in textual embeddings. The final text feature is a 16-dimensional vector suitable for lightweight classification.

(3) Graph structure feature extraction module: An interaction graph is constructed based on users’ social connections. While we adopt the DeepWalk strategy [26] to obtain initial node embeddings, we enhance this representation by further integrating structural properties. Specifically, we compute three classical centrality measures—degree, closeness, and betweenness centrality [35,36,37]—to quantify users’ topological importance. To capture the intrinsic structural patterns more effectively, we apply UMAP [34] to reduce the dimensionality of both node embeddings and centrality vectors. The final graph-based feature vector is formed by concatenating these reduced representations, enabling a comprehensive integration of local structural embeddings and global influence metrics for improved user modeling.

(4) Data feature fusion and decision module: The semantic, structural, and metadata embeddings are fused via vector concatenation to form a joint multi-source representation. We then feed this vector into the CatBoost classifier [31], a gradient boosting framework that employs ordered target statistics and symmetric tree structures to improve performance and reduce overfitting. We train the model in an end-to-end fashion using 10-fold cross-validation to evaluate classification performance.

### 3.2. Metadata Feature Extraction

In the task of social bot detection, metadata feature extraction plays an important role. For each Twitter user, in addition to the published text and friend relationship, we focus on extracting user metadata and subdivide it into account attributes, behavioral attributes, and social attributes, and define them as $M_{u}=[A_{u},B_{u},S_{u}]\in {\mathbb{R}}^{32}$ to comprehensively depict the basic characteristics and behavioral patterns of users. The following can be seen in Table 1:

(1) Account attributes: record the user’s basic information and account settings, provide a preliminary basis for understanding the user’s background and identifying the account type, and effectively distinguish ordinary users from bots.

(2) Behavioral attributes: involve the user’s activity frequency and interaction characteristics, reflect the user’s activity degree and behavior law on the platform, and reveal abnormal signals in the operation mode.

(3) Social attributes: reflect the user’s interaction and social structure in the social network and can show the closeness and location distribution of the connection between the user and other accounts.

Building on previous feature extraction approaches [23,38], we further design 10 novel metadata indicators (denoted with ‘*’ in Table 1) that capture fine-grained behavioral dynamics and social interaction patterns. These new features are motivated by observations in user behavior not addressed in prior work and are tailored to better distinguish bots from human users.

After normalization and imputation, we construct a 32-dimensional metadata feature vector, which encodes both classical and novel indicators. This representation serves as a foundational user embedding, supporting downstream modules such as text-based and graph-based detection. The combination of established and newly proposed features demonstrates both continuity with prior research and our technical innovation.

### 3.3. Text Feature Extraction

We extract historical tweet texts from a large number of Twitter accounts in the public TwiBot-22 dataset. All retweets and quote Tweets are filtered out, and only the original tweet content is retained. The remaining text is processed using the DistilBERT tokenizer [25], which transforms each tweet into a sequence of tokens in the following format

(1) $$s=[[CLS],x_{1},x_{2},\ldots ,x_{n},[SEP]],$$

where $x_{t}\in \mathbb{Z}$ is the vocabulary index of the *t* token, [CLS] is a special classification token used by DistilBERT to aggregate sentence-level representations, and [SEP] is a separator token indicating the end of the input sequence. The length of the sequence is unified to *L_max_* by dynamic padding and truncation, and the sequence after word segmentation is input to the DistilBERT encoder, which iteratively updates the text representation through the 6-layer Transformer block. The output of the L-th is represented as

(2) $$H^{\left(L\right)}=LayerNorm(H^{\left(L-1\right)}+MultiHeadAttn(H^{\left(L-1\right)})),$$

where H^1^ denotes the output of the first Transformer layer, MultiHeadAttn is the multi-head self-attention mechanism, and LayerNorm is the layer normalization operation.

Next, we use the hidden state of the last layer’s [CLS] token as the textual representation, since the [CLS] token is specifically designed in DistilBERT as a classification token. The hidden state of this token aggregates global information from the entire sequence via the self-attention mechanism:

(3) $$h_{\left[CLS\right]}^{\left(6\right)}=H^{\left(6\right)}[0,:]\in {\mathbb{R}}^{768},$$

where H^(6)^ represents the hidden state matrix of all tokens in the 6th layer of DistilBERT, and each row corresponds to a token’s contextual embedding. As a lightweight alternative to BERT, DistilBERT consists of 6 Transformer layers and outputs 768-dimensional embeddings per token. The [CLS] token is automatically inserted at the beginning of each input sequence, and is designed to aggregate global contextual information through multi-head self-attention. For k tweets from the same user, we obtain k such [CLS] vectors and compute their average as the user’s semantic-level representation,

(4) $$f=\frac{1}{k}\sum_{i=1}^{k}h_{\left[CLS](i\right)}^{\left(6\right)}\in {\mathbb{R}}^{768},$$

where *f* is the averaged textual representation that encodes the user’s overall semantic behavior. Through the forward propagation of DistilBERT encoder, each tweet is dynamically converted into a semantic embedded representation. The complete representation of a user is defined as $T=[f_{0},f_{1},\ldots ,f_{n}]$, laying the data foundation for subsequent dimensionality reduction analysis and multi-dimensional feature fusion. Although we employ DistilBERT for tokenization and text embedding, we use the model in its pre-trained form without fine-tuning on domain-specific Twitter data, considering factors such as computational cost and the generalizability of its pretrained knowledge. This design choice balances performance and efficiency, though we acknowledge that domain-specific fine-tuning could enhance model performance in future work.

### 3.4. Feature Extraction of Graph Structure

**Figure 2:**
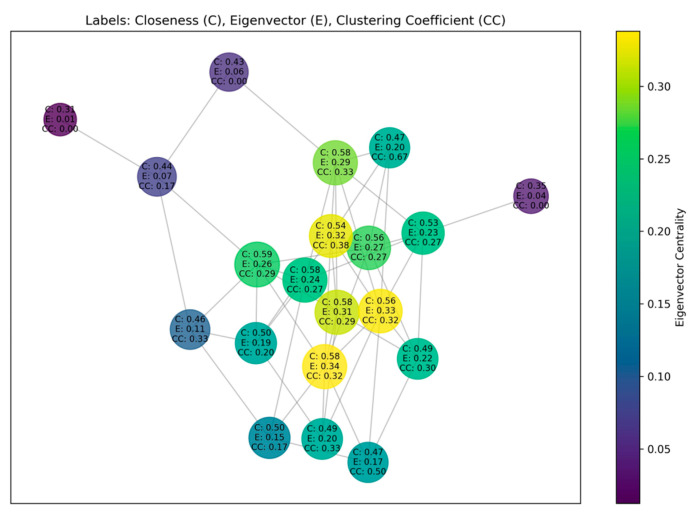
Multi-dimensional topological representation of user relationships with centrality metrics and clustering patterns.

The topological properties of social networks, as illustrated in Figure 2, are utilized to ascertain the global structural position of user nodes and local propagation behavioral patterns. These are captured synchronously through the joint optimization of quantitative analysis of structural statistics and deep wandering embedding learning, thereby offering multi-level topological evidence for bot detection.

#### 3.4.1. Global Location Extraction

Firstly, the directed relationship graph G = (V, E) is constructed, and the core metrics for nodes $v_{i}\in V$ are calculated. Degree centrality is typically used to indicate the degree of direct connection between nodes and other nodes. The number of followers and attention received can effectively reflect this. Meanwhile, betweenness centrality is mainly used to measure the role of nodes as a bridge for transmitting information between different communities. However, since the nodes in this study’s dataset have been divided by community, the role of this indicator is relatively weak. Consequently, this study has identified three distinct categories of indicators for further examination.

Closeness Centrality [35]: a metric that calculates the mean shortest path distance between a node and a network node.

(5) $$C_{c}\left(v_{i}\right)=\frac{N-1}{\sum_{v_{j\neq v_{i}}}d\left(v_{i},v_{j}\right)}.$$

Eigenvector Centrality [36]: this metric serves to evaluate the extent to which a node exerts influence over the entire network, taking into account the impact of its significant neighbors.

(6) $$A\cdot E=\lambda_{\max}\cdot E,$$

where A is the adjacency matrix of the graph, E is the eigenvector, and *λ_max_* is the largest eigenvalue.

Clustering Coefficient [37]: indicates the tightness of the connection between neighboring nodes.

(7) $$CC(v_{i})=\frac{2T(v_{i})}{\deg(v_{i})\times (\deg(v_{i})-1)},$$

where T(v_i_) is the number of triangles of node v_i_.

#### 3.4.2. Local Propagation Mode Learning

In order to capture the propagation pattern of the user population, the DeepWalk algorithm is employed to generate node embedding vectors $z_{i}\in {\mathbb{R}}^{128}$. These vectors are then obtained through random walk sequences. Furthermore, a step size of *λ_(u)_* is implemented, along with a window size of *δ_(u)_*. The Skip-gram model is utilized for optimization purposes. The objective function is designed to maximize the probability of co-occurrence of context nodes as follows

(8) $$\underset{z}{\max}\frac{1}{T}\sum_{i=1}^{T}\sum_{j\in window(i)}\log p(v_{j}|z_{i}),$$

where $z_{i}$ is the embedding vector of node $v_{i}$, $v_{j}$ is the context node, window(i) is the set of context nodes within the sliding window centered at $v_{i}$, with a window size of *δ_(u)_*. Then the index {C, CC, E} is concatenated with the embedded vector *z* and input the downstream detection module after standardization:

(9) $$h_{i}=Normalize([C(v_{i}),CC(v_{i}),e(v_{i})||z_{i}]))$$

### 3.5. Feature Fusion

In order to alleviate the dimensional disaster caused by high-dimensional feature fusion, we adopt UMAP to reduce the dimension of text embedding $T\in {\mathbb{R}}^{N\times 768}$ and node embedding $h\in {\mathbb{R}}^{N\times 128}$, respectively [39]. UMAP is a nonlinear dimensionality reduction algorithm based on manifold learning, which aims to preserve the local and global structure of data. Its core idea is to map high-dimensional data to low-dimensional spaces through fuzzy topology and probabilistic optimization [34]. First, the data needs to be standardized to eliminate the effects of different feature scales.

(10) $$X_{\mathrm{scaled}}=\frac{X-\min(X),}{\max(X)-\min(X)+\varepsilon },$$

where min(X) and max(X) are the minimum and maximum values of each feature, respectively, ε = 1 × 10^−5^, to prevent division by zero. Then for each sample $x_{i}$, calculate the local connected probability of k nearest neighbors as

(11) $$\mu_{ij}=\exp(-\frac{\max(0,d(x_{i},x_{j})-\rho_{i})}{\sigma_{i}}),$$

where $d(x_{i},x_{j})$ is the distance between $x_{i}$ and $x_{j}$, $\rho_{i}$ is the nearest neighbor distance, and the parameter $\sigma_{i}$ is usually solved iteratively such that

(12) $$\sum_{j}\mu_{ij}\approx \log_{2}(k).$$

The fuzzy set thus constructed represents the local structure of the high-dimensional data. Then in the lower dimensional space, we need to find an embed $y_{i}\in {\mathbb{R}}^{16}$ that makes the similarity distribution between the lower dimensions as consistent as possible with the fuzzy set $\mu_{ij}$ in the higher dimensional space. To do this, the probability distribution in the lower dimensional space is defined as

(13) $$v_{ij}=\frac{1}{1+a{\left\|y_{i}-\left.y_{j}\right\|\right.}^{2b}},$$

where the parameters a and b control the effect of distance on similarity, and these two parameters are usually estimated automatically by UMAP. Next, UMAP optimizes low-dimensional embedding by minimizing the cross-entropy between high–low dimensional distributions, whose objective function is typically

(14) $$L=\sum_{i\neq j}\left[\mu_{ij}\log\frac{\mu_{ij}}{v_{ij}}+(1-\mu_{ij})\log\frac{1-\mu_{ij}}{1-v_{ij}}\right].$$

By minimizing the above objective function, UMAP maps high-dimensional data X to low-dimensional embedding Y, capturing the global data distribution while preserving the local structure. In our implementation, three key hyperparameters are configured: the number of neighbors N_(p)_, the minimum distance between embedded points M_(p)_, and the output dimensionality D_(p)_. These settings are designed to balance the preservation of local neighborhood relationships and the compactness of the low-dimensional representation.

Based on the resulting embeddings, we extract both the text embedding $f_{i}^{\prime }\in {\mathbb{R}}^{N\times 16}$ and node embedding $h_{i}^{\prime }\in {\mathbb{R}}^{N\times 19}$ after dimensionality reduction, and finally fuse the semantic embedding vector after dimensionality reduction, composite graph features, and metadata feature representation into joint feature vectors, defined as $\psi_{i}=\{M_{i}||f_{i}^{\prime }||h_{i}^{\prime }\}\in {\mathbb{R}}^{N\times 67}$, to provide powerful data support for the subsequent framework decision.

### 3.6. CatBoost Classification

In order to optimize the fusion efficiency of multi-source heterogeneous features, this paper uses CatBoost to make the final classification decision. In order to prevent information leakage, CatBoost adopts ordered target coding based on sample order when processing category features. The joint $\psi_{i}$ feature vector is encoded within the formula as follows

(15) $$TE(\psi_{i})=\frac{\sum_{j:order(j)<order(i)}y_{i}+\alpha }{\sum_{j:order(j)<order(i)}1+\beta },$$

where *α* and *β* are smoothing parameters, order represents the order of samples in training, and uses historical sample information to generate feature statistics, effectively avoiding the target leakage problem in traditional mean coding. After this transformation, the original class features are mapped to numerical features, and combined with the smoothing process of a posteriori probability, making the subsequent model training more stable. CatBoost uses the gradient boosting tree algorithm to achieve the binary classification task by iteratively optimizing the following objective functions

(16) $$L(F)=-\sum_{i=1}^{N}\left[y_{i}\log(\frac{1}{1+e^{-F(x_{i})}})+(1-y_{i})\log(1-\frac{1}{1+e^{-F(x_{i})}})\right],$$

where the $1/(1+e^{-F(x_{i})})$ is the sigmoid function, which maps the model output $F(x_{i})$ to the prediction probability. In the model initialization phase, the initial prediction is usually set as the logarithmic probability of the positive example in the training set

(17) $$F_{0}(x)=\log(\frac{p}{1-p}),$$

where p is the ratio of positive cases. In each iteration, the negative gradient is first calculated as the residual to avoid the prediction deviation caused by the full data dependence of the traditional GBDT, the formula is

(18) $$g_{i}=y_{i}-\frac{1}{1+e^{-F(x_{i})}}.$$

At the same time, the second derivative can be obtained by using the sigmoid function:

(19) $$h_{i}=\frac{1}{1+e^{-F(x_{i})}}(1-\frac{1}{1+e^{-F(x_{i})}}).$$

In the process of decision tree construction, the optimal splitting point is determined by second-order Taylor expansion, and the splitting gain formula is

(20) $$Gain=\frac{1}{2}\left[\frac{({\sum_{i\in I_{L}}g_{i})}^{2}}{\sum_{i\in I_{L}}h_{i}+\lambda }+\frac{({\sum_{i\in I_{R}}g_{i})}^{2}}{\sum_{i\in I_{R}}h_{i}+\lambda }-\frac{({\sum_{i\in I}g_{i})}^{2}}{\sum_{i\in I}h_{i}+\lambda }\right].$$

where *λ* is the regularization parameter, *I_(x)_* represents the sample set of the current node, and I_L_ and I_R_ are the sample set of the left and right child nodes, respectively. For each leaf node, the leaf weights are calculated using the Newton update method, and the formula is

(21) $$\gamma_{j}=\frac{\sum_{x_{i}\in R_{j}}g_{i}}{\sum_{x_{i}\in R_{j}}h_{i}+\lambda }.$$

Finally, set the learning rate $\eta_{\left(c\right)}$ to update the output T(x) weight of the new tree into the current model:

(22) $$F(x)\leftarrow F(x)+\eta_{\left(c\right)}\cdot T(X).$$

After several rounds of iterative optimization, the above process continuously reduces the value of the objective function, and finally realizes the efficient prediction of the binary classification task.

### 3.7. Global Structure

In Algorithm 1, tweet data serve as the input. In Step 2, a 32-dimensional metadata feature vector is extracted through a series of heuristic-based computations. In Step 3, a 768-dimensional textual embedding is derived using the DistilBERT language model. Step 4 employs the DeepWalk algorithm to learn a 128-dimensional structural representation (node embedding) for each user. Subsequently, Steps 5 through 9 compute three categories of centrality metrics—namely, closeness centrality, eigenvector centrality, and clustering coefficient—which are concatenated with the node embeddings to enhance structural characterization. To address the issue of high dimensionality, UMAP is applied to jointly reduce the dimensionality of the textual and structural embeddings. The resulting low-dimensional representations are fused via vector concatenation with the metadata features to form a unified feature vector, which is subsequently fed into a CatBoost classifier for model training and prediction.
**Algorithm 1:** Bot detection via CB-MTE Frameworkinput: Twitter bot detection dataset T = {u_1_, u_2_, …, u_n_}output: Predicted labels $F(x_{i})\in$ (0: human, 1: bot)1:**for** each user $u_{i}$ in T **do**2:  metadata feature extraction: $M_{i}=f_{meta}(u_{i})\in {\mathbb{R}}^{32}$
3:  textual feature extraction: $f_{i}\in {\mathbb{R}}^{768}$ ← Equations (1)–(4)4:  Compute structural features for user $u_{i}$:5:    $C(u_{i}),CC(u_{i}),e(u_{i})$ ← Equations (5)–(7)6:    graph feature extraction: 7:      $z_{i}=DeepWalk(u_{i})\in {\mathbb{R}}^{128}$ ← Equation (8)8:    Concatenate with DeepWalk embedding:9:      $h_{i}$ ← Equation (9)10:  Reduce dimensionality via UMAP:11:  $f_{i}^{\prime }\in {\mathbb{R}}^{16},h_{i}^{\prime }\in {\mathbb{R}}^{19}$ ← Equations (10)–(14)12:  fused via vector concatenation: $\psi_{i}=concat\{M_{i},f_{i}^{\prime },h_{i}^{\prime }\}\in {\mathbb{R}}^{N\times 67}$
13:  predict label using CatBoost classifier: $F(x_{i})$ ← Equations (15)–(22)14:**end for**

## 4. Experimental Results and Analysis

### 4.1. Dataset Preparation

The dataset used in this article is TwiBot-22, the largest and most comprehensive Twitter bot detection benchmark to date [40]. Due to the massive amount of data in TwiBot-22, five sub-datasets are used for implementation in this paper. This paper evaluates each sub-dataset experimentally, as shown in Table 2.

### 4.2. Experimental Setup

#### 4.2.1. Data Preprocessing

In the research, this paper uses user data from the TwiBot-22 dataset and extracts the following three types of information: user metadata, user tweet information, and user friend relationship information. These data provide the necessary features and background information for framework training and bot user detection. In order to evaluate the performance and generalization ability of the framework more comprehensively, the 10-fold cross-validation method is used for framework validation in this paper. In the process of cross-validation, the dataset is divided into 10 mutually exclusive subsets, each containing different user samples, ensuring that the users in the training set and the validation set are completely independent to avoid data leakage.

For tweet data, this paper adopts the preprocessing methods of word segmentation, removal of stop words, and filtering of special symbols. Firstly, the word_tokenize function in the nltk library is used to segment the English tweets. This method effectively splits the text into individual words and is able to recognize punctuation and other grammatical features. Next, stop words (e.g., common English words like ‘the’ and ‘and’), which usually do not carry useful information in the text analysis, are removed. In addition, in order to avoid the influence of noise in the analysis process, special symbols in the text (such as emojis, punctuation symbols, etc.) are filtered to ensure the purity of the analysis results.

#### 4.2.2. Experimental Parameters

We provide our hyperparameter settings in Table 3 to facilitate reproducibility.

#### 4.2.3. Baseline Method: We Compare CB-MTE with the Following Baselines

- BGSRD [41] combines BERT pre-training model and graph convolutional network (GCN) BGSRD model by constructing heterogeneous graphs that integrate text semantic and social relations and jointly training them.
- Botometer [42] uses more than 1000 features derived from user metadata, content, and interactions.
- BotRGCN [7] builds heterogeneous graphs from Twitter networks and employs graph convolutional networks for user representation learning and Twitter bot detection.
- SimpleHGN [43] achieves superior performance on the heterogeneous graph benchmark HGB by building a multi-graph neural network structure that fuses node features with heterogeneous information.
- Lee et al. [44] realize efficient bot detection across social media platforms by extracting statistical features from user metadata and using a lightweight logistic regression model.
- Deshmukh et al. [45] propose a social bot detection model that integrates GraphSage and BERT, enhancing detection accuracy by fusing graph structure and textual features, and demonstrates outstanding performance in experiments.
- RGT [46], which stands for Relational Graph Converter, models the inherent heterogeneity in the Twitter domain to improve Twitter bot detection.
- SGBot [47] extracts features from the user’s metadata and feeds them into a Random Forest classifier for scalable and generalizable bot recognition.
- T5 [48] achieve state-of-the-art performance across a range of Natural Language Processing tasks by unifying all NLP tasks into a text-to-text format, and by pre-training and fine-tuning on large amounts of unlabeled data.
- BotDGT [49] is a hybrid model combining GNNs and Transformers, enabling dynamicity-aware detection of evolving social bots.

#### 4.2.4. Evaluation Indicators

In order to evaluate the framework performance, the following commonly used classification evaluation indicators are used: $Accuracy=\frac{TP+TN}{TP+TN+FP+FN}$, $Precision=\frac{TP}{TP+FP}$, $Recall=\frac{TP}{TP+FN}$, $F1=2\frac{Precision\times Recall}{Precision+Recall}$. Here, TP (true positive) represents the number of social bots correctly detected, FN (false negative) represents the number of social bots incorrectly detected, and FP (false positive) represents the number of human users incorrectly detected. TN (true negative) is the number of human users correctly detected.

### 4.3. Experimental Results and Comparative Analysis

In this paper, five sub-datasets of TwiBot-22 are trained through the CB-MTE framework proposed in this paper, and the results are shown in Table 4.

The results in Table 4 show that CB-MTE shows excellent cross-scenario detection capability on the five balanced sub-datasets of TwiBot-22: TwiBot_3 has a moderate scale of tweets and edges, and the framework achieves the highest accuracy of 0.856 and F1 score of 0.847, indicating that the moderate scale of social interaction data is conducive to multi-source feature fusion. The TwiBot_2 sub-dataset has the largest number of edges, but the framework still maintains a higher recall of 0.785, which verifies the ability of multi-source interaction module to parse dense social relations. From an overall perspective, the average accuracy of CB-MTE is 0.8214, the precision is 0.7924, the recall is 0.8254, and the F1 score is 0.8084 on the five sub-datasets, which demonstrates its consistency and stability across multiple sub-datasets. This performance verifies the effectiveness of CB-MTE in multi-source heterogeneous feature fusion, and it can accurately identify bot users in complex social media data, especially in the detection task of bot accounts, which has strong robustness.

This paper evaluates CB-MTE against ten representative baseline models on the TwiBot-22 dataset, as shown in Table 5, and the experiments show the following:

The CB-MTE framework comprehensively outperforms all baseline methods in terms of accuracy, recall, and F1 score, improves the F1 score by 23.34 percentage points over the current optimal graph model BotRGCN, and achieves a 120.8% relative improvement in F1 score over the traditional feature engineering method SGBot. This breakthrough performance stems from the deep interaction mechanism between metadata, text semantics, and social graph features, proving the effectiveness of multi-source heterogeneous feature fusion for the detection of complex adversarial scenarios.

In the graph neural model population, BotRGCN and RGT significantly outperform static metadata methods (such as SGBot) and plain text methods (such as T5), confirming that social relationship topology is the key clue for bot detection. However, the single graph structure model is limited by data sparsity, and its recall is 35.74% lower than that of CB-MTE, which highlights the necessity of multi-source complementarity.

The F1 score of models based on manual features (e.g., SGBot), are generally ineffective in new bot detection, and their average F1 score is 11.5% lower than that of the graph model. This indicates that traditional feature engineering is difficult to cope with feature camouflage enabled by large language models, while CB-MTE significantly improves its feature anti-jamming ability through the collaboration between dynamic semantic parsing and social propagation pattern mining.

### 4.4. Robustness Experiment

#### 4.4.1. Noise Robustness Experiment

**Figure 3:**
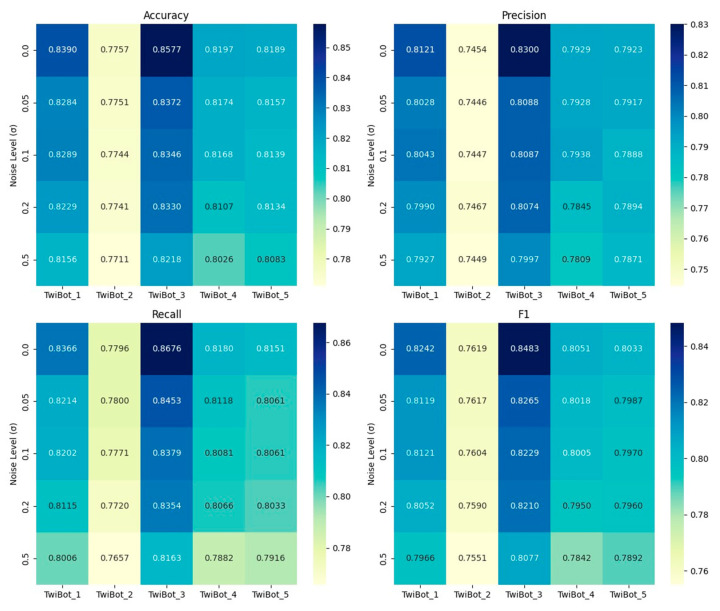
Robustness training results of the five datasets.

**Figure 4:**
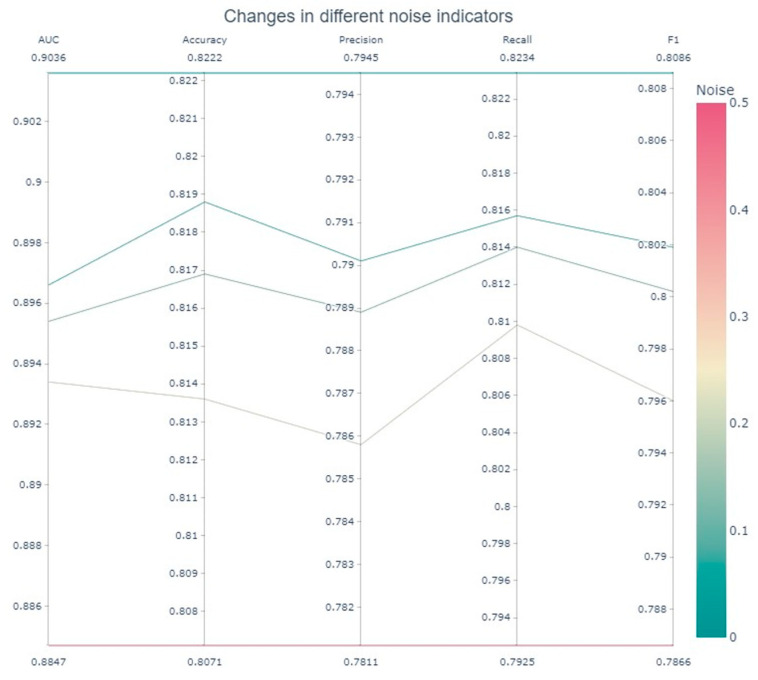
Robustness mean results—through the triple coding of color gradient (color: red → green), line width change and numerical annotation—clearly present the robustness performance of the framework under different noise levels.

To validate the robustness of the CB-MTE framework against data noise interference, this paper conducts systematic noise injection experiments on five sub-datasets of TwiBot-22. The experiment adopts the additive Gaussian noise strategy, aimed at the joint feature matrix $X\in {\mathbb{R}}^{n\times 67}$ after multi-source heterogeneous feature fusion (*n* is the number of samples, and the 67-dimensional features include 32-dimensional metadata feature + 16-dimensional text embedding + 16-dimensional graph embedding + class 3 centrality index). The noise items independently sampled by element superposition are defined as

(23) $$X_{noisy}=X+\upsilon ,\;\upsilon_{ij}∼N(0,\sigma^{2})$$

where X denotes the original feature matrix and *σ* is the standard deviation of noise, which is set to {0.05, 0.1, 0.2, 0.5} in the experiment, covering no-noise baseline (*σ* = 0), mild disturbance (*σ* = 0.05), and strong interference (*σ* = 0.5) scenarios. Under each group of noise conditions, the stability and generalization ability of the framework are evaluated through 10-fold cross-validation with the mean and standard deviation of accuracy, precision, recall and F1 score. As shown in Figure 3, heatmaps visualize the performance changes of each sub-dataset under different σ values, and Figure 4 further reveals the trend of the noise resistance of the framework through global mean analysis.

The experimental results show that CB-MTE still exhibits strong robustness under the interference of multi-dimensional noise. When the noise level increases from 0 to 0.5, the F1 score variation across sub-datasets ranges from 0.68% to 4.06%, and the variation range of accuracy is between 0.46% and 3.59%, while the F1 score of the global mean decreases by only 2.2% and accuracy by 1.51%, indicating that the multi-source heterogeneous feature fusion mitigates noise propagation through feature diversity. The recall only decreases by 3.09%, which highlights the ability of the framework to suppress missed detection; The smallest decrease in accuracy was only 1.34%, indicating that its judgment of positive samples is still robust. Although the noise resistance of the features of each module is not tested separately, the stability of fused features not only validates CB-MTE’s practicality in noisy environments, but also empirically supports multi-modal collaborative detection.

**Figure 5:**
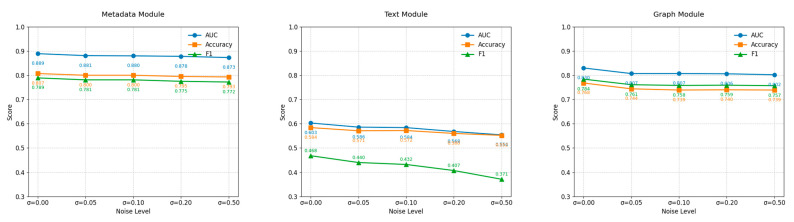
Injection of noise in different modules.

To further analyze the sensitivity of different feature modules in the CB-MTE framework to noise perturbation, this paper conducts a module-level robustness evaluation on the three types of features, namely metadata, text, and graph, based on the TwiBot_3 sub-dataset. The experiments adopt the same Gaussian noise injection strategy as that for the fused features, with the standard deviation *σ* set to {0, 0.05, 0.10, 0.20, 0.50}. Under the condition of keeping other training parameters consistent, noise perturbation tests are conducted on each type of feature separately. The performance changes of each module under the three metrics of AUC, accuracy, and F1 are shown in Figure 5.

The experimental results show that different types of features exhibit significant differences in their robustness when facing additive Gaussian noise. The metadata module shows the least performance fluctuation under various noise levels, with an AUC decrease from 0.889 to 0.873, an accuracy decrease from 0.807 to 0.793, and an F1 score decrease of only 2.2%. This demonstrates extremely strong stability. This indicates that the structured user metadata has a strong ability to resist perturbations and is insensitive to noise variations. The overall robustness performance of the graph module is second, with AUC remaining stable in the range of 0.83 to 0.802 under different noise intensities, F1 only decreasing by approximately 2.7%, and accuracy showing no significant change, always remaining between 0.768 and 0.739, indicating that the graph structure features have a better ability to maintain information when facing local noise perturbations. In contrast, the Text module is the most sensitive to noise, with the most significant performance decline. As the noise level increases from 0 to 0.50, AUC drops sharply from 0.603 to 0.554, accuracy decreases from 0.584 to 0.552, and F1 value drops from 0.468 to 0.371. This result reflects that text features are affected by the high dimensionality and sparsity of semantic representation, and are easily disrupted by noise, destroying their word vector structure, resulting in a weakened model semantic discrimination ability.

In conclusion, the module-level robustness assessment results further confirm the effectiveness of the multi-source feature fusion strategy in CB-MTE: structured information and graph structure features have inherent advantages in the noise environment, while text features need to introduce strategies such as adversarial training, noise filtering, or semantic enhancement to further improve their robustness, thereby enhancing the reliability and stability of the overall system in actual complex social environments.

#### 4.4.2. Robustness Testing of Structured Camouflage Attacks

To further verify the robustness of the proposed CB-MTE framework in real attack scenarios, we conduct a series of structured camouflage attack experiments. This attack strategy simulates the behavior of intelligent adversaries by modifying the structured features of certain bots in the test set to resemble those of normal users, thereby misleading the detection framework.

**Figure 6:**
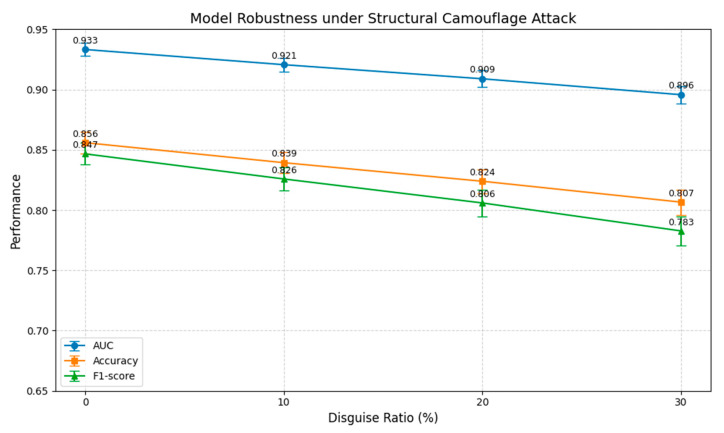
Disguise attacks at different camouflage rates.

Specifically, we use the mean and standard deviation of the structured features of human users in the training set as a reference to camouflage the samples of a specified proportion of robots in the test set. The camouflage feature vectors are generated through Gaussian sampling to simulate the behavior of “imitating normal users.” We set different camouflage proportions (10%, 20%, 30%) and evaluate the performance fluctuations of the CB-MTE framework using 10-fold cross-validation for each proportion. The performance of the framework under different camouflage proportions is shown in Figure 6.

The experimental results show that the CB-MTE framework performs well in the absence of attacks, achieving an AUC of 0.9332, an accuracy of 0.8559, and an F1 score of 0.8466, which demonstrates the effectiveness of multi-source feature integration in user behavior modeling. However, as the camouflage ratio increases, the framework’s performance exhibits a noticeable decline.

When only 10% of bot samples are camouflaged, the AUC decreases to 0.9206 (a drop of 1.3%) and the F1 score drops to 0.8258 (a decrease of 2.5%), indicating that even small-scale structural perturbations already impair the framework’s discriminative ability. As the camouflage ratio rises to 20%, the AUC and F1 scores further decrease to 0.9089 and 0.8059, respectively. When the attack intensity increases to 30%, the AUC and F1 scores drop to 0.8957 and 0.7827, suggesting that structured attacks continuously erode the framework’s detection performance.

Nevertheless, even under the highest attack intensity, the CB-MTE framework still maintains an AUC above 0.89 and an F1 score above 0.78, reflecting its inherent robustness resulting from strong feature redundancy and complementarity in the multi-source fusion process. In summary, the structured camouflage attack experiment reveals the robustness boundary of the CB-MTE framework in adversarial environments and highlights its potential vulnerability when facing adversarial samples with intelligent camouflage capabilities.

#### 4.4.3. Dimensional Analysis

**Figure 7:**
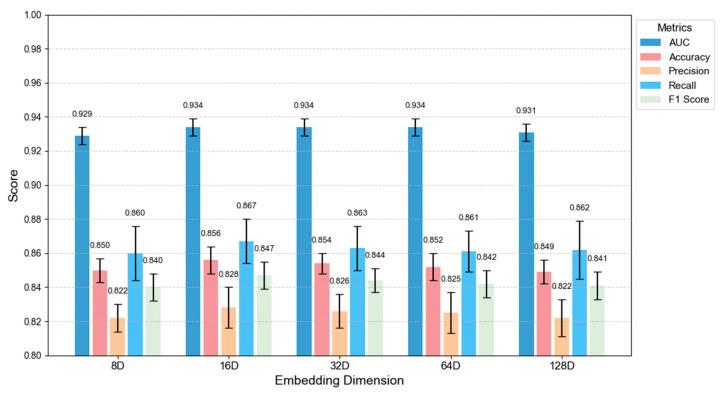
Multi-class dimension comparison diagram.

In order to systematically evaluate the impact of embedding dimension on multimodal feature fusion, this study designs parametric sensitivity experiments for textual semantic embeddings and social graph node embeddings, respectively. UMAP is used to reduce the dimensionality of two types of high-dimensional embeddings. The target dimension *d* ∈ {8, 16, 32, 64, 128}, and cross-dimensional performance comparison is conducted based on the balanced subset of TwiBot-22 dataset. Experimental results are shown in Figure 7.

This experiment reveals the robustness of the framework to feature compression through systematic comparison of multi-dimensional feature spaces: 16-dimensional embedding becomes the optimal feature representation dimension by balancing information retention and feature redundancy suppression. The experiment shows that the framework has a strong tolerance to the variation of dimensional parameters (F1 score variation < 0.6%), and the 16-dimensional embeddings achieve peak accuracy (85.6%) and recall (86.7%), which verifies the full representation ability of medium and low dimensional feature space for the behavior pattern of social bots [38]. This finding provides a theoretical basis for the design of lightweight detection framework to avoid the risk of overfitting caused by blind pursuit of high-dimensional features.

#### 4.4.4. UMAP Hyperparameter Analysis

To assess the robustness and stability of dimensionality reduction via UMAP, we conduct ablation experiments on its key hyperparameters: the number of neighbors (n_neighbors) and the minimum distance between embedded points (min_dist). The output dimensionality (n_components) is fixed at 16 for fair comparison. Table 6 presents the performance under different configurations.

We vary n_neighbors among 5, 15, and 30 to control the balance between local and global structure preservation. As shown in Table 6, all three settings yield consistently strong performance, with AUC values of 0.935 (A), 0.934 (B), and 0.935 (C), respectively. Other metrics such as accuracy, precision, recall, and F1 score also show marginal fluctuation. These results indicate that our framework is relatively insensitive to the specific value of n_neighbors, suggesting stable behavior under various local connectivity assumptions. To examine the influence of compactness in the low-dimensional space, we compare the performance under min_dist = 0.1 and min_dist = 0.5, keeping other parameters unchanged. The performance metrics between these two settings are nearly identical, with only negligible changes. This demonstrates that the model is robust against variations in min_dist and does not heavily rely on a specific embedding compactness.

The ablation results confirm that our framework maintains stable and reliable performance under different UMAP configurations, validating the robustness of our dimensionality reduction strategy.

#### 4.4.5. Classifier Selection

**Figure 8:**
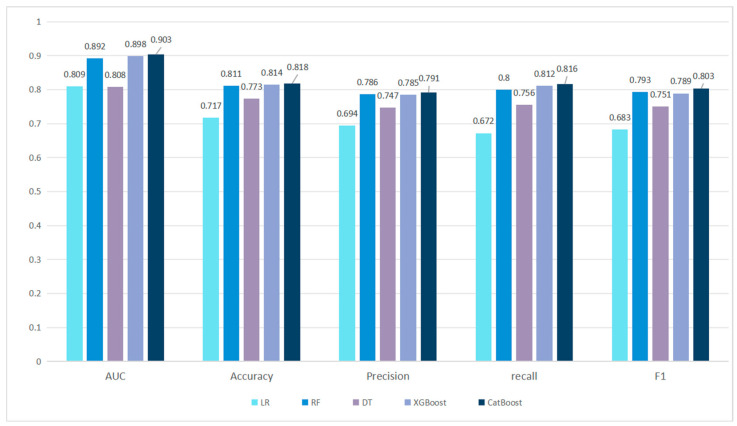
Comparison diagram of multi-class models.

In order to select a suitable decision model, this paper systematically evaluates five types of classical machine learning models—logistic regression (LR), Random Forest (RF), Decision Tree (DT), XGBoost, and CatBoost—and conducts experiments using fusion features, as shown in Figure 8.

Cross-model comparison experiments confirm that CatBoost, with its symmetric tree structure and dynamic gradient optimization mechanism, presents significant advantages in dealing with heterogeneous features such as discrete metadata and sparse social relations. Its classification performance outperforms the traditional models across the board, and it shows the lowest performance fluctuation across datasets. The algorithm effectively balances model complexity and generalization ability through adaptive regularization strategy, and its low variance characteristics meet the strict stability requirements of real-time detection systems, providing an efficient and reliable decision engine for the engineering deployment of multi-modal fusion detection frameworks.

### 4.5. Ablation Experiment Design

**Figure 9:**
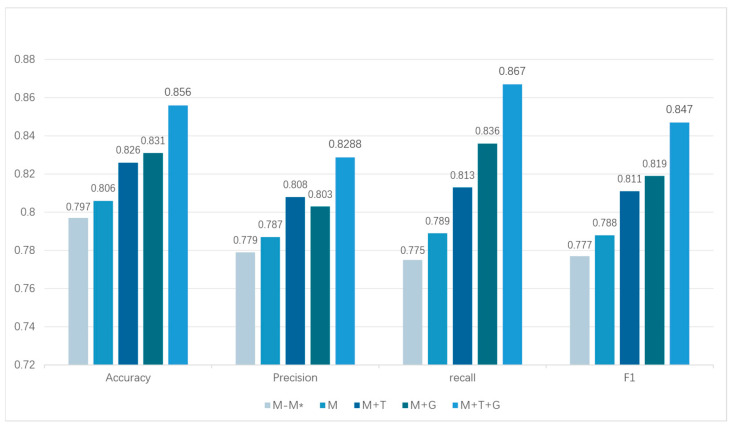
Ablation experiment.

In order to evaluate the effectiveness of the generation features of each module in the framework, ablation experiments are conducted in this paper. Based on the evaluation results of the TwiBot-22 sub-dataset in Table 4, TwiBot_3 is significantly ahead of other sub-datasets in four core indicators, with outstanding comprehensive performance and balanced indicators, which could better help identify which modules contributed the most to the overall performance. Therefore, the TwiBot_3 dataset is used for experimental evaluation of different feature combinations. Four different ablation settings were designed in the experiment, as shown in Table 7:

Through multidimensional ablation experiment, this study verified the synergistic enhancement mechanism of heterogeneous feature fusion on social bot detection, as shown in Figure 9. Specific conclusions are as follows:

Validation of the effectiveness of innovative features: Following the removal of the 10 metadata metrics (M − M*) proposed in this paper, the framework accuracy decreases from 0.806 to 0.797, the F1 score decreases from 0.788 to 0.777, and the recall decreases from 0.789 to 0.775. This phenomenon suggests that novel metadata metrics, such as the device entropy, are critical for the dynamic camouflage behavior of adversarial bots to discriminative power, which is further supported by the fact that the removal operation also causes a decline in precision and recall. This finding indicates that the novel feature system enhances the framework capacity to concurrently verify all elements and ensure accuracy through the cross-dimensional anomaly association detection mechanism.

Single-modal limitation analysis: Although the single metadata model (*M*) presents an apparent balance between recall and accuracy, its F1 score in the adversarial subset has a significant gap of 7.5% compared with the full-modal framework, revealing the inherent defects of traditional detection methods relying on static single-dimensional data, especially in the face of adversarial feature obfuscation attacks.

Multimodal enhancement effect: After the metadata feature is fused with the text feature (*M + T*), the recall increases by 3.0% and the F1 score increases by 2.9%, confirming that the natural language feature can effectively identify the text style anomaly of the generative bot. After the metadata feature is fused with the topological feature (*M + G*), the recall increases by 5.9% and the F1 score increases by 3.9%, which highlights the advantages of social topological analysis for detecting large-scale bot clusters.

Multi-source collaboration advantage: The proposed CB-MTE framework (*M + T + G*) achieves an F1 score of 0.847, which is 7.5% higher than the single-mode benchmark, and the difference between accuracy and recall is reduced to 3.8 percentage points. This performance improvement is due to the metadata–text–graph topology triple check mechanism. The cross-verification of the three forms a dynamic defense system, which makes the framework robust to multi-source counterattack.

The experimental results show that the text feature expands the detection boundary of the framework under different attack scenarios by quantifying the content generation pattern deviation and the graph structure feature by analyzing the social relationship topological anomaly. The collaborative fusion of metadata and multi-source features significantly improves the generalization ability of the system to the new adversarial bot through the cross-dimensional inconsistency detection.

## 5. Conclusions

In order to address the limitations of single-source feature characterization and the challenges of multimodal feature camouflage faced by the bot detection task in social networks, this paper proposes a CB-MTE detection framework based on the heterogeneous fusion of metadata, text, and graph topology. The paper’s contributions can be seen in three aspects: theoretical innovation, method optimization, and practical validation. (1) At the theoretical level, we construct a behavioral-semantic-topological defense system to capture hardware and behavioral timing anomalies through dynamic metadata metrics (e.g., device entropy, mutation rate of tweets, etc.), extract context-sensitive semantic features based on lightweight DistilBERT to identify generative text disguises, and analyze social topology anomalies by combining graph embedding algorithms to form a multi-dimensional joint defense system. (2) On the technical level, we propose a feature-model bi-level fusion framework, which utilizes UMAP nonlinear dimensionality reduction to eliminate the cross-source dimensionality gap between text, graph topology, and metadata, and integrates CatBoost Gradient Boosting Tree and Ordered Objective Coding to achieve efficient decision-making, with an F1 score improvement of 23.34 percentage points over the single-source graph model BotRGCN. (3) In terms of application, experiments on the TwiBot-22 benchmark set demonstrate that the discrepancy between precision and recall of CB-MTE diminishes to 3.3 percentage points, with an accuracy of 82.14%, which is notably superior to all baseline models in Table 5. On the adversarial subset TwiBot_3, CB-MTE achieves an F1 score of 84.7%, representing a 317.8% relative improvement over T5 (20.27%), which verifies the strong adaptability of the framework to complex scenarios.

This paper reveals the enhancement effect of multi-feature fusion on social entity modeling at the theoretical level, provides a scalable solution for false account identification in social network environments at the technical level, and confirms the advantages of CB-MTE in detection performance and adversarial robustness at the application level. Future research should focus on breaking through the key technologies such as heterogeneous feature fusion, lightweight graph computation, and adversarial sample defense to establish an intelligent detection system adapted to the complex ecology of social networks. In addition, the structural principles of embodied intelligent systems in robotics offer promising inspiration for extending CB-MTE. Recent developments in autonomous transportation robots and adaptive impedance control systems [50,51] highlight the benefits of modular perception–action coupling, real-time environmental adaptation, and task-aware control, which could inspire future improvements to the robustness and adaptability of social bot detection frameworks. Drawing on such architectural parallels, CB-MTE may evolve into a more generalizable and interactive detection paradigm capable of self-adjustment and deployment in dynamic, adversarial online ecosystems.

## Figures and Tables

**Table 1:** Metadata feature introduction.

Feature_Type	Symbol	Description
Account Attributes: A_u_	$E_{total}$	The total number of devices used by the user
	$H_{device}$ *	$\mathrm{Device}\;\mathrm{entropy}:-\sum p_{i}logp_{i}$, (*p_i_*)
	V	Whether user verifies: V∈ {0, 1}
	$L_{bio}$	Profile character length
	$L_{nick}$	Nicknameccer length
	$L_{user}$	Username character length
Behavior Attributes: B_u_	T	Total number of tweets
	$I_{tweet}$	$\mathrm{Tweet}\;\mathrm{audience}\;\mathrm{Index}:\;\frac{T}{F_{e}}$
	C_col_	Total of Favorites
	$R_{tweet/act}$ *	$\mathrm{Tweet}\;\mathrm{mutation}\;\mathrm{rate}:\;\frac{T}{D_{act}},(D_{act}$: active days)
	$R_{col/act}$ *	$\mathrm{Collection}\;\mathrm{mutation}\;\mathrm{rate}:\;\frac{C_{cal}}{D_{act}}$
	$R_{com/orig}$ *	$\mathrm{Original}\;\mathrm{tweet}\;\mathrm{comment}\;\mathrm{rate}:\;\frac{C}{T_{orig}}$
	$R_{com/tweet}$	$\mathrm{Tweet}\;\mathrm{comment}\;\mathrm{ratio}:\frac{C}{T},\;(C:\;$comment count)
	${\bar{L}}_{tweet}$	$\mathrm{Average}\;\mathrm{tweet}\;\mathrm{length}:\;\frac{\sum \bar{T}}{T},(\bar{T}:\;$tweet character count)
	$S_{sim}$	Content similarity of tweets
	$S_{sim/day}$ *	Single day tweet similarity
	$R_{media}$	$\mathrm{Media}\;\mathrm{rate}:\;\frac{M}{T},(M$: media count)
	$H_{time}$ *	Tweet time distribution entropy
	${\bar{T}}_{month}$	Average number of tweets per month
	$D_{recent}$	Number of days since last tweet
	$R_{url}$	$\mathrm{Link}\;\mathrm{rate}:\;\frac{L}{T},(L:\;Link\;\mathrm{coun}$)
Social attributess: S_u_	$F_{o}$	Number of follows
	$F_{e}$	Number of followers
	$I_{attn}$	$\mathrm{Follow}\;\mathrm{the}\;\mathrm{index}:\;\frac{F_{o}}{F_{o}+F_{e}}$
	$I_{pop}$	$\mathrm{Popularity}\;\mathrm{index}:\;\frac{F_{e}}{F_{o}+F_{e}}$
	$R_{mut}$ *	$\mathrm{Inverse}\;\mathrm{attention}\;\mathrm{rate}:\;\frac{M_{mut}}{F_{o}},(M_{mut}$: mutual followers)
	$R_{fo/act}$ *	$\mathrm{Mutation}\;\mathrm{rate}\;\mathrm{of}\;\mathrm{attention}:\;\frac{F_{o}}{D_{act}}$
	$R_{fe/act}$ *	$\mathrm{Fan}\;\mathrm{mutation}\;\mathrm{rate}:\;\frac{F_{o}}{D_{act}}$
	$I_{interact}$	$\mathrm{Intensity}\;\mathrm{of}\;\mathrm{engagemen}\;R+L_{k},(R:\;$$\mathrm{retweets},\;L_{k}:\;$likes)
	$R_{fwd/like}$ *	$\mathrm{Retweet}\;\mathrm{like}\;\mathrm{rate}:\;\frac{R}{L_{k}}$
	$R_{fwd/tweet}$	$\mathrm{Retweet}\;\mathrm{rate}:\;\frac{R}{T}$
	$R_{like/tweet}$	$\mathrm{Likes}\;\mathrm{rate}:\;\frac{L_{k}}{T}$

**Table 2:** TwiBot-22 sub-datasets.

	TwiBot_1	TwiBot_2	TwiBot_3	TwiBot_4	TwiBot_5
Human	5000	5000	5000	5000	5000
Bot	5000	5000	5000	5000	5000
User	10,000	10,000	10,000	10,000	10,000
Tweet	1,156,640	1,333,018	1,138,480	1,151,362	1,142,717
Edge	1,535,397	1,924,616	1,508,054	1,511,824	1,526,627

**Table 3:** Configuration of experimental parameters.

Module	Parameter Name	Parameter Size
DistilBERT	Sequence length L_max_	128
DeepWalk	Number of random walks э	100
	Random walk step λ*_(u)_*	10
	Window size δ*_(u)_*	5
CatBoost	CatBoost learning rate η_(c)_	0.03
	CatBoost iterative training times	500
	CatBoost tree depth	6
UMAP	n_neighbors N_(p)_	15
	min_dist M_(p)_	0.1
	n_components D_(p)_	16

**Table 4:** Evaluation results of TwiBot-22 sub-dataset.

	TwiBot_1	TwiBot_2	TwiBot_3	TwiBot_4	TwiBot_5	Average
Accuracy	0.8380	0.7770	0.8560	0.8180	0.8180	0.8214
Precision	0.8100	0.7450	0.8280	0.7880	0.7910	0.7924
Recall	0.8390	0.7850	0.8670	0.8200	0.8160	0.8254
F1	0.8240	0.7640	0.8470	0.8040	0.8030	0.8084

**Table 5:** Comparison of detection performance of CB-MTE based on TwiBot-22 dataset.

Model	TwiBot-22			
	Accuracy (%)	Precision (%)	Recall (%)	F1 (%)
BGSRD [41]	0.7188	0.2255	0.1990	0.2114
Botometer [42]	0.4987	0.3081	0.6980	0.4257
BotRGCN [7]	0.7966	0.7480	0.4680	0.5750
SimpleHGN [43]	0.7672	0.7257	0.3290	0.4544
Lee et al. [44]	0.7628	0.6723	0.1965	0.3041
Deshmukh et al. [45]	0.7462	-	-	0.5169
RGT [46]	0.7647	0.7503	0.3010	0.4294
SGBot [47]	0.7508	0.7311	0.2432	0.3659
T5 [48]	0.7205	0.6327	0.1209	0.2027
BotDGT [49]	0.7933	0.7242	0.4846	0.5815
**CB-MTE**	**0.8214**	**0.7924**	**0.8254**	**0.8084**

**Table 6:** UMAP different parameter results.

Number	n_neighbors	min_dist	n_components	AUC	Accuracy	Precision	Recall	F1
A	5	0.1	16	0.9350	0.8550	0.8290	0.8630	0.8460
B	15	0.1	16	0.9340	0.8560	0.8270	0.8670	0.8470
C	30	0.1	16	0.9350	0.8570	0.8290	0.8670	0.8470
D	15	0.5	16	0.9350	0.8560	0.8270	0.8670	0.8460

**Table 7:** Ablation experiments for CB-MTE.

Ablation Settings	Representation
w/o graph & text & M*	M − M*
w/o graph & text	*M*
w/o graph	*M + T*
w/o text	*M + G*
CB-MTE	*M + T + G*

## Data Availability

Restrictions apply to the availability of these data. Data were obtained from Shangbin Feng and are available https://github.com/LuoUndergradXJTU/TwiBot-22 (accessed on 31 October 2024) with the permission of Shangbin Feng.
